# Editorial for the Special Issue on Semiconductor Infrared Devices and Applications

**DOI:** 10.3390/mi12091069

**Published:** 2021-09-02

**Authors:** A. G. Unil Perera

**Affiliations:** Department of Physics & Astronomy, Georgia State University, Atlanta, GA 30303, USA; uperera@phy-astr.gsu.edu

Infrared radiation (IR) was accidentally discovered in 1800 by the astronomer Sir William Herschel. While trying to study the visible light spectrum and energy in each component, he discovered a type of invisible radiation in the spectrum that was lower in energy than red light. The thermometer used in his experiment can be considered the very first infrared detector, which is categorized as a thermal detector. The first intentional infrared detector is the thermopile developed by Macedonio Melloni in 1835 [1]. One of the early semiconductor materials used as an infrared device was lead sulfide (PbS) [2]. After the second World War, the interest in infrared devices dramatically increased as it became clear that infrared could be used to obtain images of objects due to their heat emission. A detailed history of infrared detector development is presented by Anthony Roglaski in a review article [3]. This led to the establishment of dedicated research facilities for developing infrared detectors such as the Royal Radar Establishment in Malvern, now the Qinetic in the UK, and the US Army Night Vision Laboratory, now the Night Vision and Electronic Sensors Directorate (NVESD) in VA. This focus on infrared imaging for defense applications fueled the rapid development of the field especially for the three atmospheric windows of short-wave IR (SWIR 0.7–2 µm), mid-wave IR (MWIR 3–5 µm), and long-wave IR (LWIR 8–14 µm), where the atmosphere is relatively transparent. The room temperature (300 K) black body radiation happens to peak at 10 µm, which is in the LWIR range.

For a long time, the most studied infrared detector material was HgCdTe, which was heavily used in military applications for night vision, remote sensing, and infrared astronomy research. Changing the Cd composition allowed for the detector to cover the full wavelength spectrum, covering all three ranges from SWIR and MWIR to LWIR. However, the operating temperature of 77 K was a concern for cost-conscious applications. More recently, after the development of novel thin film growth techniques such as molecular beam epitaxy (MBE) and metal–organic chemical vapor deposition (MOCVD), other materials were studied as possible infrared detectors. These developments led to the fast-paced development of the quantum well [4,5], the quantum dot [6,7], and Type II superlattice [8,9] detectors covering various material systems and multiple wavelength ranges. Most of these types of detectors were specifically geared towards various wavelength ranges and specific material systems were developed for each type of detector. Other detection principals based on physics were also studied [10,11], covering a wider wavelength range and with the possibility of being used with any material, which will be advantageous for materials that are already developed. More recently, the idea of extending the accepted standard wavelength threshold governed by the equation $\lambda_{t}=\frac{1.24}{\Delta \;}$ was demonstrated [12]. Here, λ_t_ is the threshold wavelength limit in µms and ∆ is the energy gap in meV. All the above-mentioned detector types are known as photon detectors, which are generally much faster than the detectors categorized as thermal detectors. However, thermal detectors [13] have a broad spectral range and cost advantage, making them useful for most practical applications for which millisecond response times are acceptable. Bolometers [14], which belong to the thermal detectors, have also undergone further development due to the advent of microbolometers. Recent technological advances allowing for microstructure designs have improved the response time of novel microbolometers, providing cost-effective and reasonably fast infrared detectors for mass production applications. The strong optical absorption in human tissue can, in general, be a limitation for optical imaging used for medical diagnosis. This absorbed energy leads to the thermal expansion of tissue, which can generate ultrasound energy when detected by a transducer and produce images of optical absorption contrast within tissues, now known as photoacoustic imaging [15]. A wide variety of infrared detectors have provided application opportunities for almost all areas of humanity involving security and defense, biomedical, commercial, industrial, and scientific research. In fact, the very first technique for checking COVID-19 used a near IR thermometer, which can detect body temperature without contact, providing a safe, quick, and easy way to measure the body temperature.

This Special Issue has seven papers covering various aspects of photon detection techniques. Three papers (the first, third, and fifth in this Special Issue) focus on Type II superlattice (SL) infrared detectors. In antimonide-based III–V materials grown on the GaSb substrate, the epi layers (grown on the GaSb substrate) can be lattice-matched or strained. For example, one can grow a thick layer of bulk InAs_0.91_Sb_0.09_ alloy, which is lattice-matched to GaSb with no strain in the layer. Similarly, an InAs_0.91_Sb_0.09_/GaSb superlattice also has no strain in the constituent layers as they all are lattice-matched to the GaSb substrate. Conversely, one can design a superlattice with two constituent layers, which are not lattice-matched but maintain the overall strain in the superlattice layer at zero. These are called ‘strained’ layer superlattices (SLS) because the individual layers are strained (tensile or compressive). For example, in a Ga-free InAs/InAs_(1-x)_Sb_x_ superlattice, the InAs layer is tensile-strained, while the InAs_(1-x)_Sb_x_ layer is compressively strained. The alloy composition (x) and layer thickness are the two parameters used to balance the strain in the superlattice unit cell. Similar to InAs/InAsSb superlattices, InAs/InGaSb superlattices are also strained. All the Sb-based superlattices are not technically strained (e.g., InAs_0.91_Sb_0.09_/GaSb); however, all the commonly used superlattices to date are strained. The first paper in this Special Issue, by Raphael Müller et al. from the Fraunhofer Institute, discusses the performance comparison of an InAs/GaSb Type II SL IR detector with the HgCdTe detector in a real-time spectroscopic application [16]. Comparison of roughly a decade of progress (of Type II IR detectors) with more than a half century of progress in HgCdTe IR detectors itself gives an indication of the rapid advances in the III–V-material-based infrared devices. The second paper, by Ru Chen et al. of China University of Petroleum in Beijing, discusses a manganite-based perovskite-type oxide heterojunction showing ultraviolet-to-near-infrared photo response up to room temperature [17]. Perovskite-type oxides are complex metal oxides with important applications as electrical, magnetic, and catalytic materials. The third and fifth papers here are based on InAs/InAsSb SLS structures by Gamini Ariyawansa et al. from the Air Force Research Laboratory [18] in Dayton, Ohio, and David Ting et al. from NASA JPL. Ariyawansa et al. discusses a mid-wavelength infrared detector and a focal plane array for high-temperature operations, utilizing the *n*B*n* architecture in their SLS. The fifth paper from David Ting et al. from JPL provides a discussion on the emergence of the Type II SLS infrared detectors and discusses the advantages, disadvantages, and recent developments [19]. In the last two decades, IR detectors are being specifically introduced in biomedical imaging. The fourth paper is from Hasan Göktaş et al. from Harran University in Turkey and discusses the limits of thermal sensing for microbolometers, proposing a method to improve the thermal sensitivity [20]. The sixth paper by Rayyan Manwar et al. from the University of Illinois in Chicago discusses a novel imaging technique that combines the benefits of optical resolution and acoustic depth of penetration, denoted as photoacoustic imaging [21]. The last paper of the volume, by Hemendra Ghimire et al. from the Georgia State University, discusses the concept of a heterojunction infrared detector that can be used with any semiconductor material [22]. They also describe a possible approach to detect longer threshold wavelengths beyond the corresponding energy thresholds, giving rise to the possibility of higher operating temperatures for longer wavelength detectors. Overall, this volume covers the well-developed fast response HgCdTe photon detectors to the more recently developed Type II SLS detectors, reasonably fast microbolometer thermal detectors, up and coming photoacoustic detection and imaging, and concludes with a detection technique using a novel intriguing idea of going beyond the very well-established energy gap threshold wavelength rule.
